# The Condition of Subjective Daytime Sleepiness and Its Related Decline in Work Productivity Among Daytime Workers

**DOI:** 10.2188/jea.JE20240295

**Published:** 2025-06-05

**Authors:** Yuta Takano, Toshiyuki Hirasawa, Yuichi Inoue

**Affiliations:** 1 Department of Somnology, Tokyo Medical University, Tokyo, Japan; 2 Japan Somnology Center, Neuropsychiatric Research Institute, Tokyo, Japan

**Keywords:** daytime workers, sleep duration, sleepiness, work productivity, working hours

## Abstract

**Background:**

Few have examined the condition of subjective daytime sleepiness in workers and its relation to their work productivity. This study aimed to clarify the association between the presence of subjective daytime sleepiness and work productivity measures, including presenteeism and absenteeism, as well as factors related to the presence of the symptom in daytime workers.

**Methods:**

This cross-sectional study included 17,963 daytime workers who attended the annual medical check-up. They were categorized into four groups; the daytime sleepiness group was defined as having only subjective daytime sleepiness, the insomnia group as having only insomnia symptoms, the combination group as having both subjective daytime sleepiness and insomnia symptoms, and the healthy group as having no sleep complaints. This study used demographics, health status, workplace, work productivity, and sleep items included in the self-reported medical check-up questionnaire.

**Results:**

The combination group had significantly worse presenteeism than other groups. The daytime sleepiness and insomnia groups had significantly worse presenteeism than the healthy group. The results of absenteeism were the same as presenteeism. Factors related to the positivity for subjective daytime sleepiness were presence of psychiatric disease, the positivity for habitual snoring and/or witnessed apnea, shorter sleep duration on workdays, long working hours, female sex, living alone, the amount of social jetlag, and younger age.

**Conclusion:**

Subjective daytime sleepiness, not just insomnia symptoms, has a significant negative impact on work productivity, and both workplace and individual approaches should not be ignored for addressing subjective daytime sleepiness among daytime workers.

## INTRODUCTION

Epidemiological studies have estimated that the prevalence of subjective daytime sleepiness among the general population is 7.9–33%.^1^^–^^4^ These studies also showed that numerous factors, including sociodemographic, health status, and sleep-related factors, contribute to subjective daytime sleepiness.^1^^–^^4^ In a study of truck drivers, subjective daytime sleepiness increased the risk of motor vehicular accidents independent of insufficient sleep and obstructive sleep apnea (OSA).^5^ A meta-analysis also showed that subjective daytime sleepiness increases the risk of work-related injury.^6^ Additionally, subjective daytime sleepiness reduces cognitive performance,^7^^,^^8^ which may lead to the elevated risk of job error, regardless of the job type. Thus, subjective daytime sleepiness may expose workers to potential hazards; however, its associated factors in daytime workers have not been examined, and research about workplace factors is lacking.

Worker-specific outcomes have been focused on work productivity, with several studies reporting that insufficient sleep and insomnia symptoms worsen work productivity.^9^^–^^13^ Particularly, studies have focused on insomnia symptoms and work productivity, reporting that improvement of insomnia symptoms increases work productivity.^14^^,^^15^ In contrast, only a few studies have reported the impact of subjective daytime sleepiness on work productivity^16^^,^^17^; moreover, these studies mainly targeted individuals with OSA. Subjective daytime sleepiness is a common symptom of OSA, occurring in 32.9–33.5% of patients with the disorder.^16^^,^^17^ However, subjective daytime sleepiness is related not only to OSA but also to issues with the circadian rhythm^18^^,^^19^ and insufficient sleep,^1^^–^^3^ and insufficient sleep is a serious problem among the working-age population.^20^^,^^21^ Insomnia is often associated with hyperarousal, which can be partially linked to subjective daytime sleepiness.^22^ One study found that 45.61% of patients with insomnia experience subjective daytime sleepiness.^23^ However, the mechanisms underlying subjective daytime sleepiness may vary among individuals with insomnia symptoms and subjective daytime sleepiness, subjective daytime sleepiness with OSA, and subjective daytime sleepiness without OSA. Therefore, dealing with subjective daytime sleepiness as a phenotype that can be caused by a variety of reasons and demonstrating its relationship to work productivity would improve the awareness of the potential hazard of the symptom.

Taking the above issues into consideration, this study aimed to examine the relationship between subjective daytime sleepiness and work productivity and identify the factors related to the presence of subjective daytime sleepiness among daytime workers. Particularly, our study focused on the contribution of work-related factors and sleep hygiene related issues to the presence of subjective daytime sleepiness.

## METHODS

### Procedures and participants

This study was conducted as a part of the annual medical check-up for workers registered with the Health Insurance Association for Architecture and Civil Engineering companies in Japan. The Research Ethics Committee of the Japan Depression Center determined that an ethical review was not required for the study because the data were already anonymized. This study was also approved by the Health Insurance Association for Architecture and Civil Engineering companies, Japan.

A total of 26,423 workers aged ≥18 years were included in this study, which was conducted between April 2018 and March 2019. A total of 7,524 workers were excluded due to missing responses to the following questionnaire items: presence/absence of family members living together or not (*n* = 244), working pattern (*n* = 4,207), days spent working per week (*n* = 256), number of working hours per week (*n* = 133), the score for presenteeism (*n* = 637), number of absence days in the past 4 weeks (*n* = 1,509), bedtime and wake-up time on weekdays and weekends (*n* = 387), and presence/absence of subjective insomnia (*n* = 151). Exclusion criteria for this study were shift workers and outliers for workday sleep duration. Outliers for workday sleep duration were determined by the Smirnov–Grubbs test to be less than 2 hours and greater than 10.5 hours. According to the exclusion criteria of this study, 936 workers were excluded from the study as follows: shift workers (*n* = 890) and being an outlier for workday sleep duration (less than 2 hours: *n* = 27; greater than 10.5 hours: *n* = 19). Finally, 17,963 daytime workers were included in this study.

### Measures

This study used the sociodemographic, workplace, health status, sleep, and work productivity variables. Sociodemographic variables included sex, age, and whether family members lived together or not. Workplace variables included job types (white-collar jobs: designer, sales, clerical staff, or manager; blue-collar: field staff or overseer), days spent working per week, and working hours per week (<60 hours or ≥60 hours). More than 60 hours of work per week increases the risk of cardiovascular disease,^24^ which may result in *karoshi* (ie, death from overwork). Since stress check programs for the early detection of psychiatric disease^25^ and specific health checkups and specific health guidance for the prevention of diseases caused by metabolic syndrome^26^ have been implemented to support workers’ mental and physical health in Japan, this study focused on the presence or absence of psychiatric disease and metabolic syndrome in terms of determining health status. Metabolic syndrome was defined as a case with hypertension, diabetes, and dyslipidemia.^27^ Sleep variables were the presence or absence of subjective insomnia symptoms, subjective daytime sleepiness, habitual snoring, witnessed apnea, sleep duration on workdays, and social jetlag. The participants were asked the single questions about each of their insomnia symptoms and daytime sleepiness (“Do you have insomnia symptoms?” “Do you feel daytime sleepiness?”). Social jetlag was defined as the misalignment between an individual’s circadian clock and a social clock, such as the start time of work.^28^ Presenteeism and absenteeism were used as work productivity variables. Presenteeism was defined as being present in the workplace but having decreased productivity due to health status,^29^ which was self-evaluated by participants based on their work performance over the past 4 weeks on a scale from 0% (lack of performance) to 100% (no lack of performance), with increments of 10%.^10^ Absenteeism was defined as at least 1 day of absence from work in the past 4 weeks.^10^

### Statistical analysis

Participants were categorized into daytime sleepiness, insomnia, daytime sleepiness and insomnia (combination), and healthy groups. The daytime sleepiness group was defined as having only subjective daytime sleepiness, the insomnia group as having only insomnia symptoms, the combination group as having both subjective daytime sleepiness and insomnia symptoms, and the healthy group as having neither subjective daytime sleepiness nor insomnia symptoms. Analysis of covariance (ANCOVA) was performed to compare group differences in presenteeism scores among the four groups. The Bonferroni correction was applied at the significance level for multiple comparisons. Effect sizes (Hedges’ *g*) were calculated from the *t*-values of multiple comparisons. Logistic regression analysis was performed with absenteeism as the dependent variable and the four groups as independent variables to estimate odds ratios (ORs). The covariates for both ANCOVA and logistic regression were sex (male/female), age, living condition (living alone or not), job type (white collar/blue collar), working hours per week (<60 hours or ≥60 hours), workday sleep duration, metabolic syndrome (presence/absence), psychiatric disease (presence/absence), habitual snoring and/or witnessed apnea (presence/absence), and social jetlag. “Days spent working per week” was excluded from the analyses because “working hours per week” was included in the covariates. The presence of habitual snoring and/or witnessed apnea was used for classify the presence/absence of OSA.^30^

Logistic regression analysis was performed, with subjective daytime sleepiness as the dependent variable, and sex, age, living condition, job type, working hours per week, workday sleep duration, metabolic syndrome, psychiatric disease, social jetlag, and habitual snoring and/or witnessed apnea as the independent variables, to explore the factors related to the presence of subjective daytime sleepiness. The simultaneous imputation method was used to input the variables. The daytime sleepiness, combination, and insomnia groups were compared with the healthy group to reveal the characteristics of each group.

The presence of multicollinearity, assessed using the variance inflation factor (VIF), was determined if the factor value was ≥10. Statistical significance was determined using a significance level of 5% or 95% confidence interval (CI). All analyses were performed using R 4.2.2 (R Foundation for Statistical Computing, Vienna, Austria), with the “outliers” (version 0.15), “compute.es” (version 0.2.5), “epiDisplay” (version 3.5.0.1), “performance” (version 0.10.5), and “rms” (version 6.7.1) packages.

## RESULTS

Sociodemographic, workplace, health status, sleep, and work productivity variables of daytime sleepiness, insomnia, and healthy groups are shown in Table 1. No significant difference was found in the rate of presence or absence of subjective daytime sleepiness among the months during the survey (χ^2^_[11]_ = 17.87, *P* = 0.085).

**Table 1.** Descriptive variables of the participants

	Daytime sleepiness group (*n* = 1,820)	Insomnia group (*n* = 3,213)	Combination group (*n* = 1,208)	Healthy group (*n* = 11,722)
Sociodemographic variables				
Age, mean (SD)	41.54 (12.67)	49.34 (10.74)	46.53 (12.00)	45.84 (11.83)
Male sex, *n* (%)	1,491 (81.9)	2,583 (80.4)	1,000 (82.8)	9,964 (85.0)
Living alone, *n* (%)	581 (31.9)	971 (30.2)	405 (33.5)	2,978 (25.4)
Workplace variables				
White collar, *n* (%)	1,017 (55.9)	2,033 (63.3)	757 (62.7)	6,907 (58.9)
Working ≥60 hours per week, *n* (%)	461 (25.3)	377 (11.7)	222 (18.4)	1,806 (15.4)
Days spent working per week, mean (SD)	5.27 (0.53)	5.17 (0.56)	5.2 (0.53)	5.21 (0.53)
Health status variables				
Metabolic syndrome, *n* (%)	14 (0.77)	47 (1.5)	25 (2.1)	86 (0.7)
Psychiatric disease, *n* (%)	10 (0.5)	52 (1.6)	29 (2.4)	40 (0.3)
Sleep variables				
Workday sleep duration, hours, mean (SD)	5.83 (0.99)	6.34 (1.04)	5.99 (1.01)	6.32 (0.98)
Social jetlag, hours, mean (SD)	1.16 (1.23)	0.95 (1.62)	1.11 (1.67)	0.93 (1.36)
Habitual snoring and/or witnessed apnea, *n* (%)	563 (30.9)	1,037 (32.3)	418 (34.6)	3,076 (26.2)
Work productivity variables				
Presenteeism, mean (SD)	78.73 (16.63)	81.28 (15.29)	75.89 (17.76)	85.19 (14.27)
Absenteeism, *n* (%)	195 (10.7)	407 (12.7)	198 (16.4)	974 (8.3)

SD, standard deviation.

**Figure 1.**
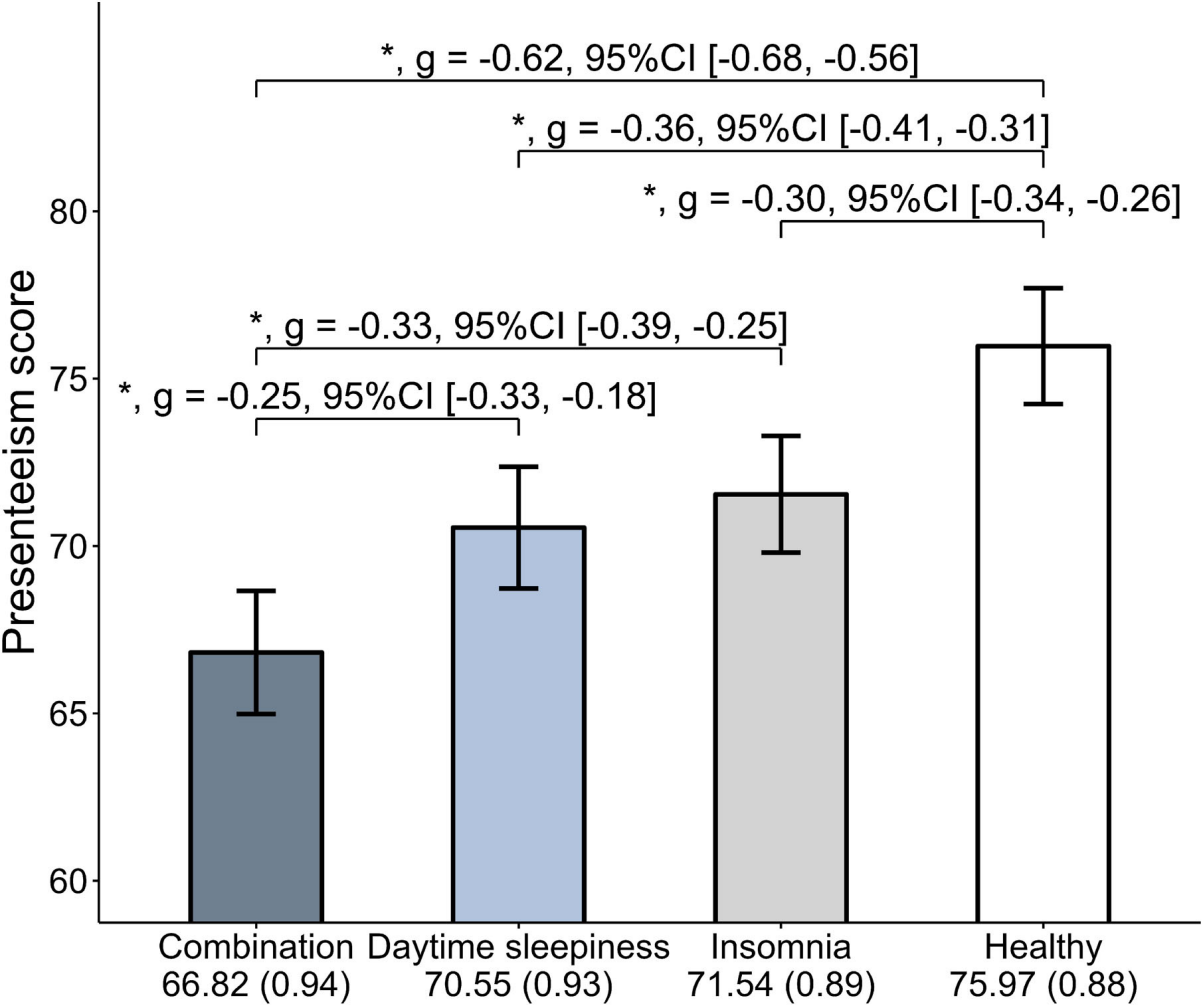
Results of analysis of covariance comparisons among four groups. Adjusted for sex, age, living alone, job types, working hours per week, metabolic syndrome, psychiatric disease, workday sleep duration, social jetlag, and habitual snoring and/or witnessed apnea. The mean values are adjusted. Values in parentheses are standard errors. The error bars represent the 95% confidence interval. ^*^P < 0.05. CI, confidence interval.

The ANCOVA results showed significant group differences in presenteeism scores (*F*_[3,17949]_ = 248.61, *P* < 0.05) (Figure 1), with the combination group having significantly lower presenteeism scores than the daytime sleepiness group (estimate −3.72; 95% CI, −5.16 to −2.29, *g* = −0.25), the insomnia group (estimate −4.72; 95% CI, −6.03 to −3.41, *g* = −0.33), and the healthy group (estimate −9.14; 95% CI, −10.32 to −7.97, *g* = −0.62). Both the daytime sleepiness group and the insomnia group had significantly lower presenteeism scores than the healthy group (daytime sleepiness group: estimate −5.42; 95% CI, −6.41 to −4.43, *g* = −0.36; insomnia group: estimate −4.42; 95% CI, −5.20 to −3.65, *g* = −0.30). The daytime sleepiness group and the insomnia group did not differ significantly in presenteeism scores. In summary, the combination group had the worst level of presenteeism, while the daytime sleepiness group and the insomnia group exhibited worse levels of presenteeism compared to the healthy group.

The results of the logistic regression analysis of the between-group comparison of absenteeism are shown in Table 2. The combination group had significantly increased odds of absenteeism compared with the daytime sleepiness group (adjusted OR 1.54; 95% CI, 1.24–1.92), the insomnia group (adjusted OR 1.38; 95% CI, 1.14–1.66), and the healthy group (adjusted OR 2.08; 95% CI, 1.76–2.46). Both the daytime sleepiness group and the insomnia groups had significantly increased odds of absenteeism compared with the healthy group (daytime sleepiness group: adjusted OR 1.35; 95% CI, 1.14–1.59; insomnia group: adjusted OR 1.51; 95% CI, 1.33–1.71). The daytime sleepiness group and the insomnia group did not show significant differences in absenteeism. Multicollinearity was not present, as the VIF ranged from 1.02–1.20.

**Table 2.** Results of logistic regression analysis of the comparison of absenteeism between groups

	Unadjusted		Adjusted	

	OR	95% CI	OR	95% CI
Combination group	2.16	1.83–2.55	2.08	1.76–2.46
Daytime sleepiness group	1.32	1.13–1.56	1.35	1.14–1.59
Insomnia group	1.60	1.42–1.81	1.51	1.33–1.71
Healthy group	ref		ref	

Combination group	1.35	1.12–1.63	1.38	1.14–1.66
Daytime sleepiness group	0.83	0.69–0.99	0.89	0.74–1.08
Insomnia group	ref		ref	
Healthy group	0.62	0.55–0.71	0.66	0.58–0.75

Combination group	1.63	1.32–2.02	1.54	1.24–1.92
Daytime sleepiness group	ref		ref	
Insomnia group	1.21	1.01–1.45	1.12	0.93–1.35
Healthy group	0.76	0.64–0.89	0.74	0.63–0.88

CI, confidence interval; OR, odds ratio; ref, reference.

Adjusted for sex, age, living alone, job types, working hours per week, metabolic syndrome, psychiatric disease, workday sleep duration, social jetlag, and habitual snoring and/or witnessed apnea.

**Figure 2.**
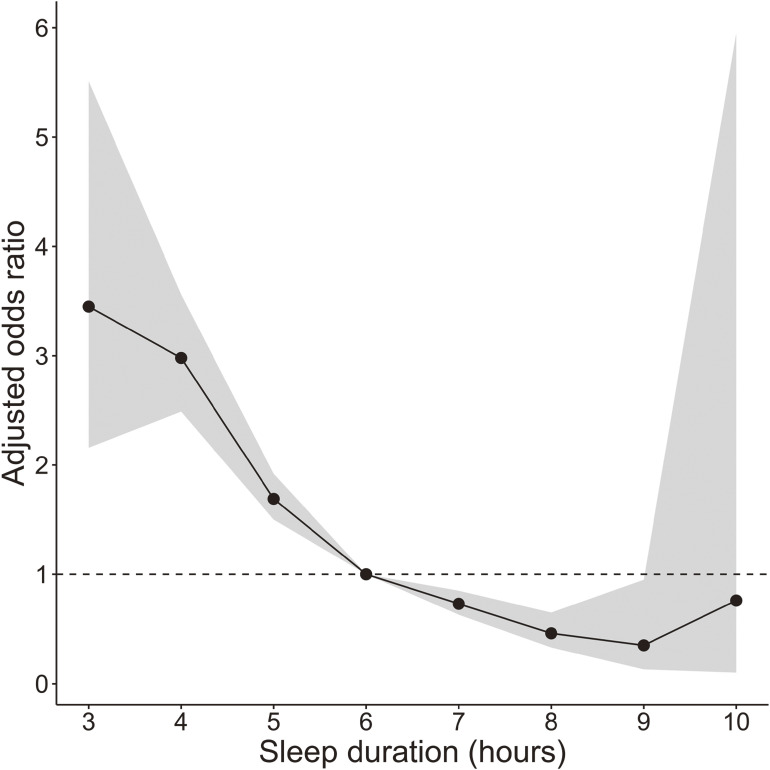
Structure of the relationship between subjective daytime sleepiness and workday sleep duration (daytime sleepiness group vs healthy group). Adjusted for sex, age, living alone, job types, working hours per week, metabolic syndrome, psychiatric disease, social jetlag, and habitual snoring and/or witnessed apnea. The dots plot is the adjusted odds ratio, and the graph range is the 95% confidence interval.

**Figure 3.**
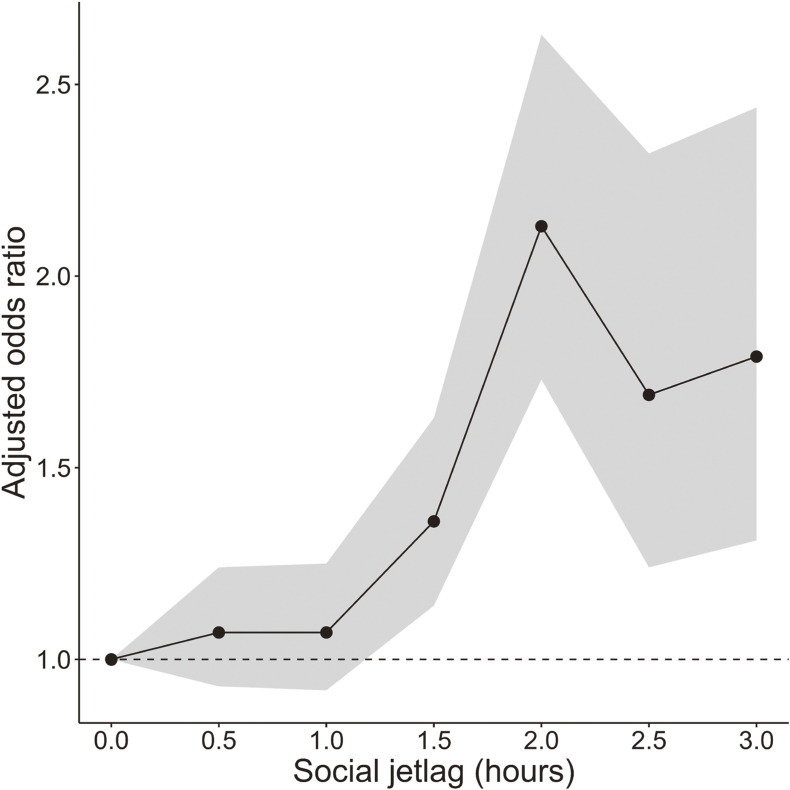
Structure of the relationship between subjective daytime sleepiness and social jetlag (daytime sleepiness group vs healthy group). Adjusted for sex, age, living alone, job types, working hours per week, metabolic syndrome, psychiatric disease, workday sleep duration, and habitual snoring and/or witnessed apnea. The dot plot is the adjusted odds ratio, and the graph range is the 95% confidence interval.

Significant factors associated with subjective daytime sleepiness were age (adjusted OR 0.97; 95% CI, 0.97–0.98), male sex (adjusted OR 0.78; 95% CI, 0.67–0.90), living alone (adjusted OR 1.21; 95% CI, 1.08–1.36), long working hours per week (adjusted OR 1.27; 95% CI, 1.11–1.45), workday sleep duration (adjusted OR 0.62; 95% CI, 0.59–0.66), presence of psychiatric disease (adjusted OR 2.20; 95% CI, 1.08–4.49), social jetlag (adjusted OR 1.06; 95% CI, 1.03–1.10), and the positivity for habitual snoring and/or witnessed apnea (adjusted OR 1.44; 95% CI, 1.29–1.62) (Table 3). Multicollinearity was not present, as the VIF ranged from 1.00–1.27. Regarding sleep duration, compared with 6 hours of sleep on workdays, less than 6 hours of sleep significantly increased the positivity for subjective daytime sleepiness. In contrast, 7–9 hours of sleep significantly decreased the positivity (Figure 2). Furthermore, the relationship between social jetlag and subjective daytime sleepiness had a non-linear structure: the positivity for subjective daytime sleepiness significantly and rapidly increased when social jetlag reached 1.5 hours, peaking at 2 hours (Figure 3). Considering the relation between the presence of OSA and subjective daytime sleepiness, the participants were stratified based on the presence/absence of habitual snoring and/or witnessed apnea. However, working hours per week and workday sleep duration were still factors significantly related to the positivity for subjective daytime sleepiness (eTable 1 and eTable 2). Multicollinearity did not exist in the results of this sensitivity analysis, as the VIF of the model with habitual snoring and/or witnessed apnea ranged from 1.00–1.25, and the VIF of the model without habitual snoring and/or witnessed apnea ranged from 1.00–1.30.

**Table 3.** Associations of descriptive parameters with the presence of subjective daytime sleepiness (daytime sleepiness group vs healthy group)

	Unadjusted		Adjusted	

	OR	95% CI	OR	95% CI
Age	0.97	0.97–0.97	0.97	0.97–0.98
Sex				
Female sex	ref		ref	
Male sex	0.80	0.70–0.91	0.78	0.67–0.90
Living condition				
Not living alone	ref		ref	
Living alone	1.38	1.24–1.53	1.21	1.08–1.36
Job type				
Blue collar	ref		ref	
White collar	0.88	0.80–0.98	1.09	0.97–1.22
Working hours per week				
<60 hours	ref		ref	
≥60 hours	1.86	1.66–2.09	1.27	1.11–1.45
Metabolic syndrome				
No	ref		ref	
Yes	1.05	0.59–1.85	1.58	0.88–2.83
Psychiatric disease				
No	ref		ref	
Yes	1.61	0.81–3.23	2.20	1.08–4.49
Workday sleep duration, hours	0.60	0.57–0.63	0.62	0.59–0.66
Social jetlag, hours	1.09	1.06–1.12	1.06	1.03–1.10
Habitual snoring and/or witnessed apnea				
No	ref		ref	
Yes	1.26	1.13–1.40	1.44	1.29–1.62

CI, confidence interval; OR, odds ratio; ref, reference.

The results of the related factors for the combination and insomnia groups compared to the healthy group are shown in Table 4. All the variables other than long working hours per week were significantly related to the combination group. Multicollinearity was not present, as the VIF ranged from 1.01–1.22. Age, female sex, living alone, presence of psychiatric disease, and the positivity for habitual snoring and/or witnessed apnea were significantly related to the insomnia group. Multicollinearity was not present, as the VIF ranged from 1.00–1.19.

**Table 4.** Associations of descriptive parameters with combination and insomnia groups (combination group vs healthy group; insomnia group vs healthy group)

	Combination group				Insomnia group			

	Unadjusted		Adjusted		Unadjusted		Adjusted	

	OR	95% CI	OR	95% CI	OR	95% CI	OR	95% CI
Age	1.00	1.00–1.01	1.01	1.01–1.02	1.03	1.02–1.03	1.03	1.03–1.03
Sex								
Female sex	ref		ref		ref		ref	
Male sex	0.85	0.72–0.99	0.76	0.64–0.91	0.72	0.65–0.80	0.59	0.53–0.67
Living condition								
Not living alone	ref		ref		ref		ref	
Living alone	1.48	1.30–1.68	1.65	1.45–1.89	1.27	1.17–1.39	1.54	1.41–1.69
Job type								
Blue collar	ref		ref		ref		ref	
White collar	1.17	1.04–1.32	1.21	1.05–1.39	1.20	1.11–1.30	1.03	0.94–1.13
Working hours per week								
<60 hours	ref		ref		ref		ref	
≥60 hours	1.24	1.06–1.44	1.16	0.98–1.38	0.73	0.65–0.82	0.91	0.80–1.04
Metabolic syndrome								
No	ref		ref		ref		ref	
Yes	2.86	1.82–4.48	2.39	1.50–3.80	2.01	1.40–2.87	1.40	0.97–2.02
Psychiatric disease								
No	ref		ref		ref		ref	
Yes	7.18	4.44–11.63	8.87	5.40–14.55	4.80	3.18–7.27	5.11	3.35–7.79
Workday sleep duration, hours	0.71	0.67–0.75	0.70	0.65–0.74	1.02	0.98–1.06	0.98	0.94–1.02
Social jetlag, hours	1.07	1.03–1.10	1.06	1.03–1.10	1.01	0.98–1.03	1.01	0.99–1.04
Habitual snoring and/or witnessed apnea								
No	ref		ref		ref		ref	
Yes	1.49	1.31–1.69	1.55	1.36–1.76	1.34	1.23–1.46	1.37	1.26–1.50

CI, confidence interval; OR, odds ratio; ref, reference.

Combination group is coexistent with subjective daytime sleepiness and insomnia symptoms.

## DISCUSSION

This study aimed to examine the relationship between subjective daytime sleepiness and work productivity and identify factors related to the presence of subjective daytime sleepiness among daytime workers. Both presenteeism and absenteeism were worst in the combination group, with both subjective daytime sleepiness and insomnia symptoms, and worse in the daytime sleepiness and the insomnia groups compared with the healthy group. Notably, this study found that the daytime sleepiness group and the insomnia group did not differ with regard to work productivity measures. Factors related to the positivity for subjective daytime sleepiness among daytime workers included presence of psychiatric disease, the positivity for habitual snoring and/or witnessed apnea, shorter sleep duration on workdays, long working hours, female sex, living alone, the amount of social jetlag, and younger age.

OSA with subjective daytime sleepiness causes worse presenteeism than OSA without subjective daytime sleepiness.^16^^,^^17^ These previous studies have suggested a risk of subjective daytime sleepiness in workers with OSA for developing presenteeism. However, the results of our study showed that subjective daytime sleepiness was associated with worse presenteeism among general daytime workers regardless of the presence/absence of OSA. Additionally, the daytime sleepiness group and the insomnia group did not differ with regard to presenteeism. The results of our study show that subjective daytime sleepiness also worsens presenteeism to almost the same as insomnia, which has been a focus in the relationship between sleep problems and presenteeism.

The relationship between insomnia symptoms and absenteeism has been reported in a previous study.^10^ Individuals with subjective daytime sleepiness reportedly have a higher rate of absenteeism.^31^ Furthermore, our study found that the relationship between subjective daytime sleepiness and absenteeism was comparable to that of insomnia and absenteeism, even after adjusting for sociodemographic, workplace, health status, and sleep variables.

The variable most closely associated with the presence of subjective daytime sleepiness was psychiatric disease. The reason for this phenomenon is unknown; however, disease-related lifestyle changes, treatment drugs, or the symptoms themselves may have played a role in the mechanism. Habitual snoring and/or witnessed apnea also had a high OR as a related factor of subjective daytime sleepiness, which is consistent with the fact that daytime sleepiness is widely accepted as an important symptom of OSA,^32^ impressing the importance of the assessment for the presence of OSA. However, the novelty of this study was that long working hours per week as a workplace factor, and social jetlag as well as sleep duration on workdays as sleep hygiene issues, were associated with subjective daytime sleepiness among the daytime workers.

Workplace-level intervention that reduce working hours per week by 25% reportedly result in increased sleep duration on workdays, decreased perceived stress, and improved fatigue and subjective daytime sleepiness.^33^ Long working hours are associated with fatigue,^34^ and fatigue is associated with subjective daytime sleepiness.^2^ Thus, long working hours result in chronic fatigue, which may appear as subjective daytime sleepiness.

Reportedly, social jetlag is associated with subjective daytime sleepiness in daytime workers.^9^ Notably, in our study, social jetlag of 1.5 hours or longer was associated with the presence of subjective daytime sleepiness, and the association reached its peak at social jetlag of 2 hours in this study. Thus, a small delay in sleep timing on days off is acceptable, but avoiding further delay (ie, a discrepancy of 1.5 or more hours between midpoints of sleep on workdays and days off) may be important for preventing subjective daytime sleepiness in daytime workers.

The association between short sleep duration and subjective daytime sleepiness is widely accepted,^1^^–^^3^ and a similar relationship was observed in this study. The evident relationship between short sleep duration on workdays and subjective daytime sleepiness, even after stratifying the participants by habitual snoring and/or witnessed apnea, which is one of the characteristics of OSA, highlighted the importance of workday sleep duration among daytime workers. The recommended sleep duration per night for the working-age population has been set at 7–9 hours.^35^ Similarly, our results showed a minimal presence of subjective daytime sleepiness at 7–9 hours of workday sleep duration in the study population, suggesting that 7–9 hours of sleep duration on workdays is recommended to prevent subjective daytime sleepiness in daytime workers. Reportedly, the relationship between sleep duration and subjective daytime sleepiness shows a U-shaped curve.^3^ However, in our study, workday sleep duration greater than 10.5 hours was excluded as an outlier, which could have contributed to the lack of the formation of a U-shaped curve in the analysis.

Younger age and living alone were significantly associated with the presence of subjective daytime sleepiness, consistent with previous studies.^2^^,^^4^^,^^36^ However, differing from previous studies,^2^^,^^3^^,^^36^ the present study showed a significant female predominance for the presence of subjective daytime sleepiness. Since the mean age of women in the analysis was 41.9 years, this finding may be partially explained by the cultural background, because Japanese women tend to sleep less than men, especially after the age of 40 years.^20^

The combination of the decline in cognitive performance due to subjective daytime sleepiness^7^^,^^8^ and the psychological distress due to insomnia symptoms^37^ in the combination group may have resulted in the worst work productivity measures, including presenteeism and absenteeism. However, it is unclear whether subjective daytime sleepiness or insomnia symptoms are the cause of impaired work productivity in this group. The combination group had numerous related factors in comparison to the healthy group. Specifically, being white-collar workers, the presence of metabolic syndrome, shorter sleep duration on workdays, and the amount of social jetlag were related factors in the combination group but not in the insomnia group. Therefore, both approaches to subjective daytime sleepiness and insomnia symptoms are necessary in the combination group, while the focus on subjective daytime sleepiness should mainly be on sleep hygiene education. Cognitive behavioral therapy for insomnia (CBT-I) is known to be effective in improving insomnia symptoms among daytime workers.^38^ Therefore, CBT-I can be considered as an approach to insomnia symptoms.

This study had some limitations. First, authorized validated scales, such as the Epworth Sleepiness Scale^39^ and Athens Insomnia Scale,^40^ were not used to assess subjects’ daytime sleepiness and insomnia symptoms, and the severity of these symptoms could not be identified. The questions used in this study to assess insomnia symptoms and daytime sleepiness did not ask about the duration of symptoms. As a result, it was not possible to determine how long participants had been experiencing these symptoms. This means that both short-term and long-term symptoms were included in the study. Second, neither authorized validated tools for OSA screening, such as the Berlin Questionnaire^41^ and STOP-Bang questionnaire,^42^ nor pulse oximetry were used in this study. The OSA screening with the item asking about habitual snoring and/or witnessed apnea used in this study had a sensitivity of 94.8% and specificity of 13.3%,^30^ possibly overestimating OSA. Third, although obesity is associated with subjective daytime sleepiness,^43^ our study could not obtain information about the body mass index of the participants. Moreover, since we did not obtain information about the medications used by the participants, we were not able to examine the effect of the medication on their subjective daytime sleepiness. Furthermore, although some psychosocial factors are associated with work productivity,^44^ we did not examine them. Fourth, since the percentage of sleep duration greater than 10 hours is rare worldwide,^45^^,^^46^ it is reasonable to consider outliers for sleep duration to be greater than 10.5 hours. On the other hand, it is noteworthy that 30.51% of the participants in this study slept less than 6 hours, indicating a significant proportion of Japanese individuals with short sleep duration on workdays compared to those in other countries.^45^^,^^46^ Therefore, it is important to be cautious when applying the results of this study to other countries. Fifth, a causal relationship between subjective daytime sleepiness and work productivity could not be determined in this cross-sectional study. Future prospective longitudinal studies should clarify the causal relationship between subjective daytime sleepiness and work productivity impairment and between subjective daytime sleepiness and its causative factors. Finally, the demographic data indicated that individuals excluded due to missing data were more likely to be older, female, and blue-collar workers compared to individuals with complete data. However, it is uncertain whether these characteristics have influenced the results of the study.

Despite these limitations, this study is the first to show that daytime workers who experience subjective daytime sleepiness have higher levels of presenteeism and absenteeism compared to healthy workers. Their levels are similar to those of workers with insomnia. Additionally, the coexistence of subjective daytime sleepiness and insomnia symptoms could have an especially negative impact on the workplace. Furthermore, long working hours, short sleep duration on workdays, and a certain level of social jetlag are associated with the presence of subjective daytime sleepiness, even after adjusting for OSA-related symptoms. The results of this study suggest that subjective daytime sleepiness has a significant negative impact on work productivity and that both workplace and individual approaches should not be ignored when addressing subjective daytime sleepiness among daytime workers.

## SUPPLEMENTARY MATERIAL

eTable 1. Descriptive variables between the participants having subjective daytime sleepiness without and with habitual snoring and/or apnea

eTable 2. Sensitivity analysis of descriptive parameters associated with daytime sleepiness
