# Supplementary material for: The Condition of Subjective Daytime Sleepiness and Its Related Decline in Work Productivity Among Daytime Workers
Source: J Epidemiol. 2025 Jun 5;35(6):262–9. doi: 10.2188/jea.JE20240295 (PMC12066196; doi:10.2188/jea.JE20240295)
Supplement: Supplementary file 1 [file je-35-262-s001.pdf]

eTable 1. Descriptive variables between the participants having subjective daytime sleepiness without and with habitual snoring and/or apnea

	Subjective daytime sleepiness without habitual snoring and/or apnea group (n=1,257)	Subjective daytime sleepiness with habitual snoring and/or apnea group (n=563)
Sociodemographic variables		
Age, years, mean (SD)	39.7 (12.87)	45.63 (11.17)
Male sex, n (%)	981 (78.0)	510 (90.6)
Living alone, n (%)	443 (35.2)	138 (24,5)
Workplace variables		
White collar, n (%)	709 (56.4)	308 (54.7)
Working ≥60 hours per week, n (%)	314 (25.0)	147 (26.1)
Days spent working per week, mean (SD)	5.26 (0.51)	5.3 (0.57)
Health status variables		
Metabolic syndrome, n (%)	9 (0.7)	5 (0.9)
Psychiatric disease, n (%)	8 (0.6)	2 (0.4)
Sleep variables		
Workdays sleep duration (h), mean (SD)	5.85 (0.99)	5.78 (0.99)
Social jetlag, hours, mean (SD)	1.26 (1.38)	0.93 (0.72)

SD, standard deviation.

eTable 2. Sensitivity analysis of descriptive parameters associated with daytime sleepiness

	Subjective daytime sleepiness without habitual snoring and/or witnessed apnea Adjusted OR (95% CI)	Subjective daytime sleepiness with habitual snoring and/or witnessed apnea Adjusted OR (95% CI)
Age, years	0.96 (0.96–0.97)	1.01(1.00–1.01)
Male sex (ref = female sex)	0.68 (0.58–0.80)	1.57 (1.16–2.12)
Living alone (ref = not living alone)	1.27 (1.11–1.45)	0.97 (0.79–1.19)
White collar (ref = blue collar)	1.11 (0.97–1.28)	1.02 (0.85–1.23)
Working ≥60 hours (ref = <60 hours)	1.25 (1.06–1.46)	1.41 (1.13–1.76)
Metabolic syndrome (ref = no)	1.94 (0.96–3.92)	1.19 (0.47–2.97)
Psychiatric disease (ref = no)	2.55 (1.16–5.60)	1.35 (0.32–5.70)
Workday sleep duration, hours	0.64 (0.60–0.68)	0.58 (0.53–0.64)
Social jetlag, hours	1.08 (1.04–1.11)	0.98 (0.91–1.06)

CI, confidence interval; OR, odds ratio; ref, reference.
